# CD26 Expression on T Helper Populations and sCD26 Serum Levels in Patients with Rheumatoid Arthritis

**DOI:** 10.1371/journal.pone.0131992

**Published:** 2015-07-15

**Authors:** Oscar J. Cordero, Rubén Varela-Calviño, Tania López-González, Cristina Calviño-Sampedro, Juan E. Viñuela, Coral Mouriño, Íñigo Hernández-Rodríguez, Marina Rodríguez-López, Bruno Aspe de la Iglesia, José María Pego

**Affiliations:** 1 Department of Biochemistry and Molecular Biology, University of Santiago de Compostela, Santiago de Compostela, Spain; 2 Service of Immunology, University Hospital Complex of Santiago de Compostela, Santiago de Compostela, Spain; 3 Service of Rheumatology, University Hospital Complex of Vigo, Vigo, Spain; 4 IRIDIS (Investigation in Rheumatology and Immuno-mediated Diseases) Group, Instituto de Investigación Biomédica. Xerencia de Xestión Integrada-SERGAS, Vigo, Spain; University of Leuven, Rega Institute, BELGIUM

## Abstract

We studied dipeptidyl peptidase IV (DPP-IV, CD26) expression in different T helper cells and serum soluble DPP-IV/sCD26 levels in rheumatoid arthritis (RA) patients, correlated these with disease activity score (DAS), and examined how they were affected by different therapies, conventional or biological (anti-TNF, anti-CD20 and anti-IL6R or Ig-CTLA4). The percentage of CD4^+^CD45R0^+^CD26^-^ cells was greatly reduced in patients (up to 50%) when compared with healthy subjects. Three other subsets of CD4 cells, including a CD26high Th1-associated population, changed variably with therapies. Data from these subsets (frequency and staining density) significantly correlated with DAS28 or DAS28 components but different in each group of patients undergoing the different therapies. Th17 and Th22 subsets were implicated in RA as independent CCR4^+^ and CCR4^-^ populations each, with distinct CD26 expression, and were targeted with varying efficiency by each therapy. Serum DPP-IV activity rather than sCD26 levels was lower in RA patients compared to healthy donors. DPP-IV and sCD26 serum levels were found related to specific T cell subsets but not to disease activity. We conclude that, according to their CD26 expression, different cell subsets could serve to monitor RA course, and an uncharacterized T helper CD26^-^ subset, not targeted by therapies, should be monitored for early diagnosis.

## Introduction

Molecular biomarkers for earlier diagnosis of rheumatoid arthritis (RA), (achieving remission of the disease is possible if diagnosed in the early stages)[1,2], and predictors of response to therapies or follow up markers of disease progression or remission are in demand [3]. Diminished dipeptidyl peptidase IV (DPP-IV, soluble CD26) activity in both serum and synovial fluid of RA patients has been previously reported [4,5] and we have also found a relationship between serum sCD26 levels and RA activity [6] (not all DPP-IV activity is ascribed to sCD26 [7]). Other groups reported similar or contradictory results in RA and other rheumatic diseases including systemic lupus erythematosus (SLE) [8–10].

Changes in DPP-IV/sCD26 levels were are also found in other diseases. Briefly, low levels of DPP-IV activity or soluble CD26 were observed in immuno-suppressed situations including some tumours; whereas high levels occur in other tumours, and infectious, inflammatory and liver diseases [11]. These qualitative or quantitative changes may be important in RA and possibly in the pathogenesis of other diseases, since DPP-IV as a result of its N-terminal X-Pro cleaving activity, regulates chemotactic responses to the inflammatory chemokines CCL, 3–5, 11 and 22, and CXCL, 2 and 9–12 [9], including SDF-1 [12,13]; In addition, it regulates other biologically active peptides such as NPY and VIP, recently implicated in RA [14].

DPP-IV (CD26) is expressed on the surface of both immune and non-immune cell types as well as the soluble molecule found in biological fluids such as serum [11,12] In addition CD26 may participate in T cell activation [15] and cell infiltration of the arthritic joints through its non-enzymatic key roles in adhesion and invasion [16,17].

While in SLE patients the number of CD26^+^ T cells decreases and this reduction positively correlates with sCD26 levels [8], others have reported the opposite behaviour in RA. It has been described [15] that patients with active disease display higher percentages of (mainly) CD4^+^ CD26^+^ T cells and higher CD26 surface density, whereas CD26 expression on synovial fluid T lymphocytes is low. Ellingsen et al [18] found that active chronic RA is characterized by enhanced CD26 density on both circulating monocytes and CD4^+^ T lymphocytes, although without significant correlation with disease activity index. Previous studies with small cohorts have shown effects of methotrexate (MTX) and anti-TNF-**α** antibody therapy (adalimumab) on CD26 density on monocytes and serum DPP-IV activity levels accompanied by improvements in the Disease Activity Score (DAS28) [18,19].

CD4^+^ T helper cells comprise multiple subsets, representing different differentiation stages and activation levels; CD26 is differentially expressed in those subsets. The CD4^+^ CD45R0^+^ CD26high population, identified as effector Th1 lymphocytes, correlates with clinical severity in multiple sclerosis [20]. It has also been reported that Th17 cells express high levels of CD26 [21], and Th17 as well as Th22 populations have been implicated in the pathogenesis of RA [14,16] since their numbers were elevated in RA patients and the percentages of both cell populations correlated positively with disease activity [22].

In order to identify early events that can be targeted with preventive or therapeutic measures; we studied the levels of serum CD26 and DPP-IV activity and its correlation with CD26 cell surface expression in T helper subsets from RA patients, grouped according to the type of therapy: conventional or biological (using immunomodulating agents).

## Patients and Methods

### Study Design

One hundred and ten patients from the Rheumatology Service (Hospital Meixoeiro-CHUVI) were recruited in a cross-sectional case-control study. Patients fulfilled the American College of Rheumatology (ACR) criteria of 1987 [1] and were on various therapies, including biological therapies (BT). A group of 25 healthy donors (women, n = 6, men, n = 19; mean age was 44 years (range: 20–62) was also recruited.

### Ethics Statement

All the procedures described were performed according to clinical ethical practices of the Spanish and European Administrations and approved by the Local Ethics Committee (Comité Ético de Investigación Clínica de Galicia, Xunta de Galicia, code 2010/298). Written informed consent was obtained from all participants.

### Assessment of Disease Activity

Disease activity was assessed by the DAS-28 index, which takes into account the number of tender joints, swollen joints, erythrocyte sedimentation rate (ESR) and Patient Global Assessment (PGA) of disease activity, scored by a numeric rating scale (NRS 0–100). Erythrocyte sedimentation rate (ESR), C-reactive protein (CRP), haemoglobin and platelet concentration levels were also recorded as RA activity markers. All patients completed the HAQ (Stanford Health Assessment Questionnaire) [23], which allows for self-reporting of disability measure.

### Biological samples

For serum collection, peripheral venous blood extracted with BD SST II *Advance* tubes was allowed to clot at room temperature and centrifuged at 2,000 x *g* for 15 min. Serum was stored at -80°C until use. Blood cells were collected using TransFix Vacuum Blood Collection Tubes (Cytomark, Buckingham, UK) and stored at 4°C until use.

### Flow Cytometry Analysis

For tetracolour flow cytometry determinations of CD26 expression on T cells, routine protocols have been used [24]. Peripheral blood mononuclear cells were stained with an optimized mix of anti-CD3/CD4/CD45R0/CD26 antibodies (20 L/10^6^ cells (Immunostep, Salamanca, Spain) in PBS containing 1% BSA and 0.05% sodium azide (FACS buffer) and incubated at 4°C for 30 min. Subsets of CD4 T cells were classified according to their expression of CD26 (i.e., CD26high, considered Th1 cells) [20, 25].

Th17 or Th22 lineages are almost exclusively CCR6^+^ [14, 26]. Whereas Th22 cells express the additional chemokine receptors CCR4 and CCR10 [16, 27, 28], Th17 cells express CD161 in addition to CCR4, [27–29]. Th17 and Th22 subsets were characterized by staining with combinations of anti-CD4-APC, anti-CD161-PE and anti-CD194 (CCR4)-PerCP-Cy5.5 (BD Pharmingen), anti-CD196 (CCR6)-FITC (eBioscience) and anti-CCR10-PE (R&D systems). The CD4^+^CCR6^+^CD161^+^CCR4^-^ subset has been recently described as non TGF-**β** secreting Th17 cells [30], in contrasts to Th17 CCR4^+^ cells, which secrete TGF-**β**; data for both of these populations together with data for the same both Th22 populations, were recorded.

Cells were acquired using a Becton-Dickinson FACScalibur and analyzed with the Flowing software program (Perttu Terho, Turku Centre for Biotechnology, Finland, EU). Viability of cells was analysed by physical parameters of size / volume and morphological complexity.

### Measurement of DPP-IV Enzyme Activity and Soluble CD26 Protein

Both techniques have been described previously [31,32]. Briefly, DPP-IV activity was measured in 96-well culture plates using Gly-Pro-p-nitroanilide (0.2 mM, Sigma-Aldrich) as substrate in reaction mixtures (100 μL) containing serum samples (10 μL) and 50 mM Tris-HCl, pH 8.0 [25,26]. After 15 min, the hydrolysis of the substrate was monitored at 405 nm wavelength using a BioRad Model 680 microplate reader. These results were standardized by a standard curve for p-nitroaniline (Sigma-Aldrich data sheet), detection limit 0.6 nmol, and the activity was expressed as IU L-1. All experiments were performed in duplicate unless otherwise specified. The sCD26 concentration was measured with the human DPPIV/CD26 DuoSet ELISA development System kit (RnD Systems) according to the manufacturer's instructions (the limit of detection specified is 20 pg/mL). Colorimetric quantification was performed with the same microplate reader at 450/540 nm.

Since previous studies with large cohorts [32,33] have shown no statistically significant differences in both levels of sCD26 and DPP-IV activity according to gender or age, values for healthy controls and RA patients were therefore not matched for gender and age.

### Statistical Analysis

All analyses were parametric. The ANOVA test was carried out to compare variables among the four groups of patients with or without biological therapies. The post-hoc Scheffé test was used for variables with homogeneous variances and the post-hoc Dunnett´s C test was used for variables without homogeneous variances. Dunnett´s t test was performed to compare each group with a control group, either the group without biological therapy or the healthy donor group. Student´s t-test was also used to compare variables between two groups. Statistical analyses were carried out using the SPSS version 21 software (SPSS, Chicago IL, USA).

## Results

### Demographic and clinical characteristics of RA patients

The 110 RA patients consisted of 82 women and 28 men. The mean age was 57 years (range: 24–78), which was not statistically different from healthy donors. Mean disease duration was 12 years (range: 0.5–41). Disease activity parameters and HAQ [23], were recorded on the same day of blood sample collection, and are listed in Table 1.

**Table 1:** Disease activity parameters of patients grouped according to their biological (BT) or conventional (no BT) therapies.

	No BT (n = 21)		Anti-TNFα BT (n = 47)		Anti-CD20 BT (n = 10)		Anti-IL6R/Ig-CTLA4 BT (n = 13)	
	Mean ± SD	CI (95%)	Mean ± SD	CI (95%)	Mean ± SD	CI (95%)	Mean ± SD	CI (95%)
**SW28**	1.54 ± 2.32	0.51–2.58	0.91 ± 1.52	0.52–1.31	2.28 ± 3.45	0.29–4.28	1.2 ± 1.86	0.17–2.23
**TEN28**	1.54 ± 3.02	0.21–2.88	0.98 ± 2.47	0.34–1.63	4.43 ± 8.07	(-)0.23–9.09	1.71 ± 2.67	0.17–3.26
**DAS28**	3.3 ± 1.1	2.8–3.8	3.4 ± 1.2	3.08–3.72	3.87 ± 1.51	2.99–4.74	2.54 ± 1.44	1.71–3.37
**PGA**	34.09 ± 22.37	23.91–44.28	39.60 ± 23.93	33.31–45.89	52.5 ± 21.73	39.95–65.04	42.33 ± 26.45	27.69–56.98
**HAQ**	0.89 ± 0.86	0.48–1.31	1.09 ± 0.71	0.90–1.28	1.59 ± 0.61	1.23–1.94	1.23 ± 0.76	0.79–1.68
**CRP (mg/L)**	8.67 ± 8.99	4.34–13.0	6.49 ± 8.8	4.05–8.94	9.4 ± 7.44	5.1–13.7	3.19 ± 3.16	1.44–4.94
**Platelets (× 10** ^**9**^ **cel/L)**	262.31 ± 107.29	214.74–309.88	262.03 ± 86.53	239.48–284.58	255.5 ± 90.2	203.42–307.58	222.53 ± 39.78	200.5–244.56
**% Erythrocytes**	40.26 ± 3.51	38.7–41.81	40.2 ± 4.3	39.08–41.32	41.6 ± 4.55	38.97–44.23	40.48 ± 4.36	37.72–43.96
**Haemoglobin (g/dL)**	13.55 ± 1.07	13.07–14.02	13.42 ± 1.48	13.03–13.80	13.68 ± 1.19	12.99–14.37	14.17 ± 1.62	13.28–15.07
**ESR (mm/h)**	30.14 ± 13.33	24.23–36.05	35.98 ± 25.35	29.38–42.59	26.64 ± 19.23	15.54–37.75	8.73 ± 8.28	4.15–13.32

n = number of patients; BT = biological therapy; CI = confidence interval; SD = standard deviation; SW28 = Swollen Joint Count; TEN28 = Tender Joint Count; DAS28 = Disease Activity Score; PGA = Patient Global Assessment; HAQ = Health Assessment Questionnaire; CRP = C-reactive protein; ESR = Erythrocyte Sedimentation Rate

Patients were on different therapies and the cohort was divided into four groups according to the mechanism of action of the therapeutic: a) No biological therapies (BT) group: patients on Disease-Modifying Anti-Rheumatic Drugs (DMARDs; methotrexate and/or leflunomide); b) anti-TNF-**α** agents (adalimumab, etanercept, infliximab, golimumab, certolizumab); c) anti-CD20 mAb (rituximab), and d) anti-IL6R mAb (tocilizumab) or Ig-CTLA4 (abatacept). In this latter subgroup both therapies showed similar responses, so patients were grouped together (data not shown).

### Cell surface CD26 in different naïve and effector/memory T cell populations in RA patients with different therapies

**Fig 1:**
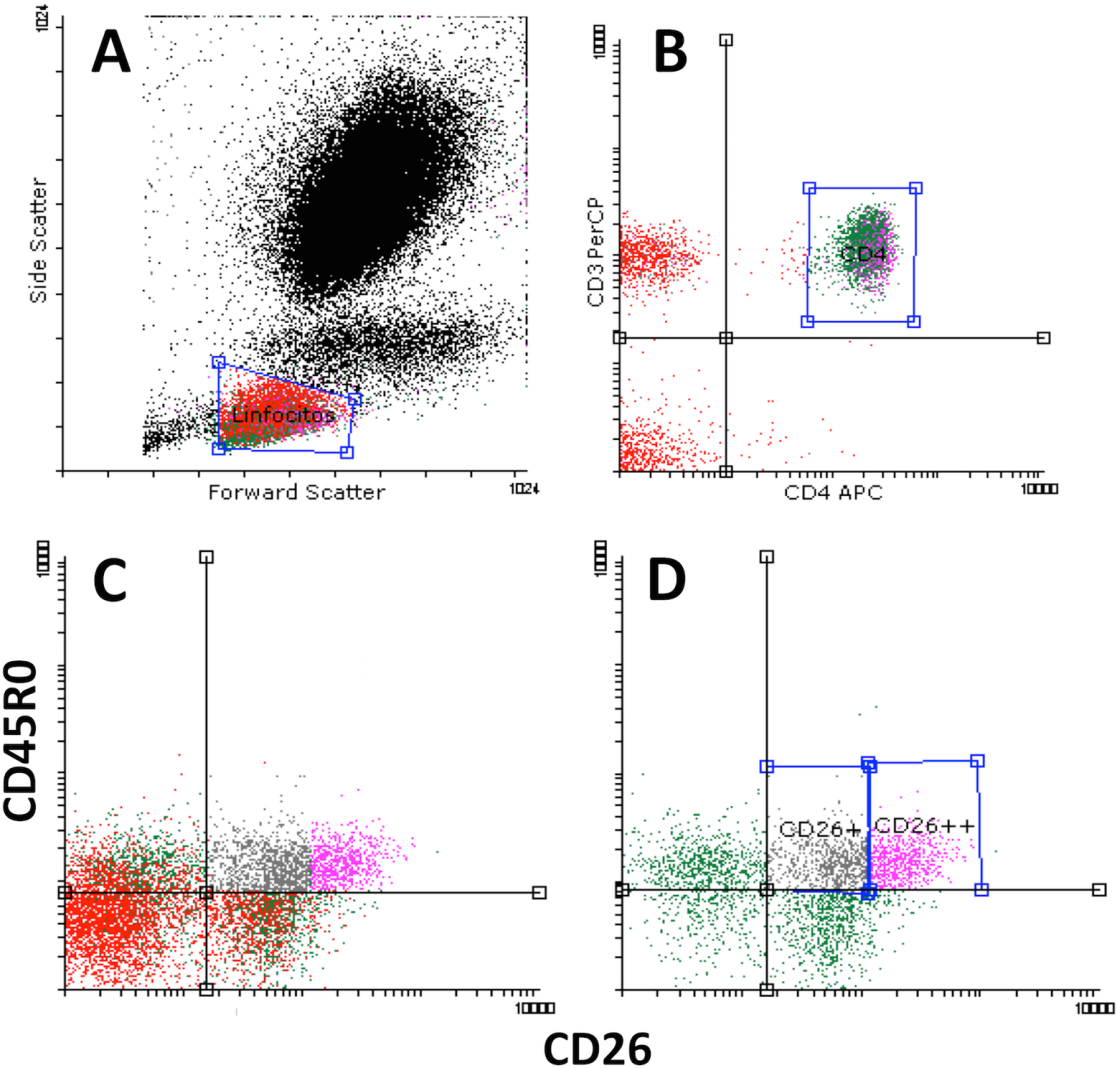
Major CD4^+^ T cell subsets defined by surface CD45R0 and CD26 expression. A) Representative flow cytometry dot-plot showing lymphocytes gated physically. B) Representative dot-plot of gated CD4^**+**^ T lymphocytes. C) and D) Dot-plots showing the differential expression of CD45R0 and CD26 in the whole lymphocyte region gated in A (C) or in the CD3^**+**^CD4^**+**^ region gated in B (D). Naïve CD4 cells are mostly CD26^**+**^ in contrast to the CD8^**+**^ cells (compare C and D). From D) four major subsets were selected by their differential expression of CD45R0 and CD26: CD4^**+**^CD45R0^**low/−**^ CD26^**+**^ (naïve T cells); and effector/memory CD4^**+**^CD45R0^**+**^CD26^**-**^ (CD26 negative), CD4^**+**^CD45R0^**+**^CD26^**+**^ (intermediate) and CD4^**+**^CD45R0^**+**^CD26^**++**^ (CD26high).

As expected [20], most naïve CD4^+^ CD45RA cells are CD26^+^. Fig 1 shows flow cytometry dot plots and how the populations were defined. The first subset, called CD45R0^-^ CD26^+^, includes CD45R0^low^ cells: these naïve T cells were CCR5^-^, CXCR3^-^, CXCR6^-^, L-Selectin^+^ and CCR7^+^ (data not shown and [20]). The effector/memory T cells (CD4^+^CD45R0^+^) were divided into three subsets according to their CD26 expression: average or intermediate (CD45R0^+^ CD26^+^, which express plasma membrane CD26 as seen in the naïve T cell population); high (CD45R0^+^ CD26^++^), and cells that did not show expression (CD45R0^+^ CD26^-^) [20]. The CD26high population was gated from the right of CD26 expression (as mean of fluorescence intensity, MFI, data) in the CD45RA population. According to this criterion, the CD26high subset expressed 3 to 6 times more CD26 that the CD26^+^ average population. CD4^+^CD45R0^+^CD26^++^ cells were, generally, CCR7^-^, L-Selectin^low^, CXCR3^+^ (80%) and in some cases were CCR5^+^ (data not shown and [20]) indicating a Th1 phenotype. CD4^+^CD45R0^+^CD26^-^ cells are more heterogeneous for these memory/effector markers, an important percentage being CXCR3^+^ or CCR7^+^ as well as the population with more CCR4^+^ cells.

Percentages and surface CD26 levels of these four cell subsets were recorded for each patient (Table 2). Comparing the RA patient cohort as a whole (n = 91) to the healthy subjects (n = 11), a slightly higher percentage of experienced (CD45R0^+^) cells, was observed (45.5 ±13.5 vs. 40.0 +/-8.8%) as well as a higher percentage of CD4^+^ CD26^+^ cells (77.0 ±11.7 vs. 70.4 ±8.6%) [15,18]. Strikingly, the major difference between both groups was found in the percentage of the CD45R0^+^ CD26^-^ population (12.5 ±6.8 of total CD4 vs. 19 ±4.8% in healthy controls). Percentages of CD45R0^+^ CD26^++^ (9.4 ±6.1 vs. 7.6 ±2.7%), CD45R0^+^ CD26^+^ (23.6 ±9.4 vs. 23.3±6.1%) and CD45R0^-^ CD26^+^ (44.1 ±13.3 vs. 45.4 ±9.9%) were very similar. Thus, it seems that it is the statistically significant diminution of the experienced CD26^-^ population (t-test p = 0.003) that largely accounts for the increment of CD4 CD26^+^ cells in RA patients.

**Table 2:** Percentages of CD4 T cell subsets gated according to the effector/memory CD45RO and CD26 staining and CD26 cell surface level (MFI) in RA patients undergoing different therapies.

	No BT (n = 21)		Anti-TNFα BT (n = 47)		Anti-CD20 BT (n = 10)		Anti-IL6R/Ig-CTLA4 BT (n = 13)	
	Mean ± SD	CI (95%)	Mean ± SD	CI (95%)	Mean ± SD	CI (95%)	Mean ± SD	CI (95%)
**% Lymphocytes**	20.64 ± 9.42*	16.35–24.92	27.91 ± 10.92* #	24.70–31.11	18.76 ± 6.38*	14.20–23.33	19.08 ± 11.69*	12.01–26.14
**% CD3** ^**+**^ **CD4** ^**+**^	45.94 ± 8.12*	42.23–49.63	47.84 ± 8.51	45.34–50.33	53.85 ± 9.97* #	46.72–60.99	48.62 ± 7.05	44.36–52.89
**% CD26** ^**+**^ **in CD4**	73.89 ± 13.5*	67.75–80.04	77.39 ± 10.51*	74.31–80.48	85.51 ± 6.6*	80.79–90.23	74.36 ± 13.21*	66.38–82.35
**% CD45RO** ^**+**^ **in CD4**	47.28 ± 15.73	40.12–54.44	46.26 ± 13.77	42.21–50.30	42.67 ± 10.02	35.50–49.83	41.95 ± 11.18	35.19–48.70
**% CD45RO** ^**-**^ **CD26** ^**+**^ **in CD4**	40.02 ± 12.75*	34.21–45.82	43.96 ± 13.75	39.92–47.99	51.98 ± 10.11*	44.75–59.22	44.21 ± 13.18	36.24–52.18
**% CD45RO** ^**+**^ **CD26** ^**-**^ **in CD4**	13.43 ± 7.8	9.88–16.98	13.05 ± 6.4	11.17–14.94	9.15 ± 5.93	4.91–13.4	11.85 ± 7.21	7.5–16.21
**% CD45RO** ^**+**^ **CD26** ^**+**^ **in CD4**	23.73 ± 8.33	19.94–27.52	24.31 ± 7.66	22.06–26.56	23.55 ± 6.86	18.64–28.46	23.21 ± 6.12	19.51–26.91
**% CD45RO** ^**+**^ **CD26** ^**++**^ **in CD4**	10.43 ± 9.5	6.1–14.75	9.5 ± 4.93	8.05–10.94	9.74 ± 4	6.87–12.6	7.11 ± 3.56	4.95–9.26
**CD26 MFI in CD45RO** ^**+**^ **CD26** ^**+**^	53.72 ± 8.35	49.92–57.52	61.19 ± 32.86	51.54–70.83	55.94 ± 5.81	51.78–60.09	52.07 ± 4.27	49.49–54.65
**CD26 MFI in CD45RO** ^**+**^ **CD26** ^**++**^	224.62 ± 40	206.4–242.82	239.45 ± 42.57	226.95–251.95	236.28 ± 37.33	209.58–262.98	227.61 ± 44	201.02–254.21
**CD26 MFI in CD45RO** ^**-**^ **CD26** ^**+**^	65.03 ± 37.87	47.79–82.27	67.9 ± 17.7*	62.70–73.10	66.88 ± 17.31	54.49–79.26	55.96 ± 14.92*	46.95–64.98

n = number of samples; BT = biological therapy; CI = confidence interval; SD = standard deviation; MFI = mean of fluorescence intensity

* Values significantly different among groups (not specified) at p < 0.05 with Student´s t-test

# Values significantly different to those of no biological therapy (No BT) group at p < 0.05 with Dunnett’s t-test

**Fig 2:**
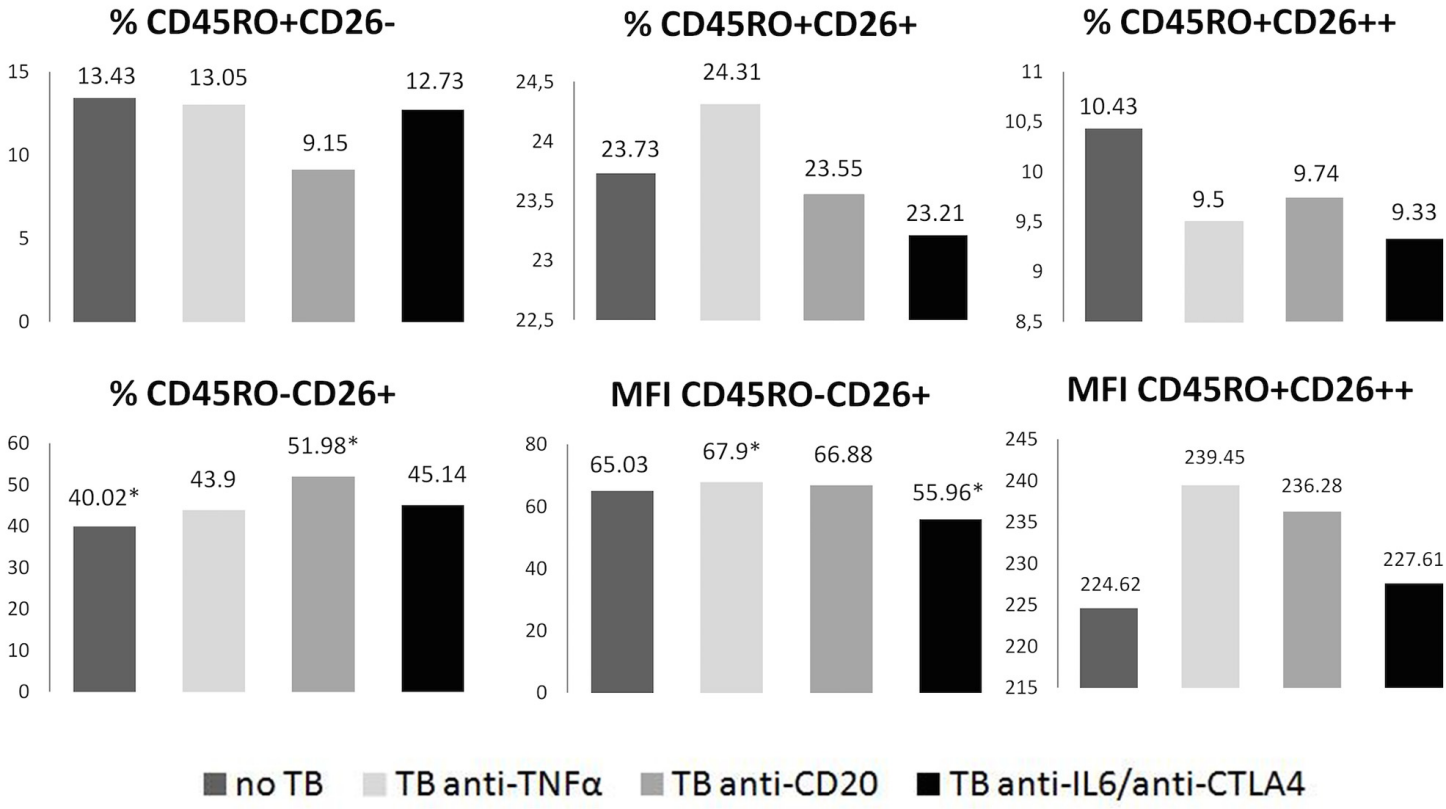
Effect of different therapies on CD4^+^ T cell subsets and cell surface CD26 levels. Frequencies of different CD4^**+**^ T cell populations defined by CD45R0 and CD26 expression, and CD26 levels (MFI) in those cell populations, were determined by flow cytometry in RA patients undergoing different therapies. Confidence interval can be found in Table 1. Asterisks show statistically significant differences (p<0.05; Student’s t-test).

Similar comparisons were performed among the different groups of patients (Table 2). The No BT group had the lowest percentages of CD45R0^-^ CD26^+^ and CD45R0^+^ CD26^-^ subsets and the higher percentages of CD26^++^ in comparison to healthy donors (Table 2). This group may resemble RA patients diagnosed before therapy [18]. Neither of the BT, at the moment of the measurements, was recovering all the values (frequencies of the subsets) of healthy controls and their effects were very different to each other: anti-IL-6R/anti-CTLA4 recovered most, and the worst results were seen for the anti-CD20 group (Table 2, Fig 2 shows some data).

The total number of cells in each lymphocyte subset was also analyzed (Table 3), and resulted in similar findings. Most remarkable, is that the number of CD26high (CD45R0^+^CD26^++^) cells was the same in all four groups of patients. The anti-TNF and anti-CD20 groups had larger CD45R0^-^CD26^+^ subsets, thereby contributing to the higher number of total CD4^+^CD26^+^ cells (around a 40% more) in RA. However, both groups showed differences in the other subsets: the anti-TNF group had more CD45R0^+^CD26^+^ cells, while the anti-CD20 group had less CD45R0^+^CD26^-^ cells.

**Table 3:** Total cell number of CD4 T cells subsets gated according to the effector/memory CD45RO and CD26 staining and CD26 cell surface level (MFI) in RA patients undergoing different therapies.

	No BT (n = 21)		Anti-TNFα BT (n = 47)		Anti-CD20 BT (n = 10)		Anti-IL6R/Ig-CTLA4 BT (n = 13)	
(x 10^9^ cel L^-1^)	Mean ± SD	CI (95%)	Mean ± SD	CI (95%)	Mean ± SD	CI (95%)	Mean ± SD	CI (95%)
**Lymphocytes**	2.11 ± 0.94	1.71–2.53	2.73 ± 0.98* #	2.47–2.99	2.06 ± 0.8**	1.6–2.53	2.47 ± 0.54**	2.01–2.62
**CD3+ CD4+**	0.99 ± 0.45	0.78–1.2	1.26 ± 0.51*	1.12–1.41	1.12 ± 0.42	0.82–1.42	1.12 ± 0.28	0.95–1.29
**CD26+ in CD4**	0.72 ± 0.35	0.57–0.89	0.97 ± 0.43*	0.84–1.1	0.96 ± 0.38	0.69–1.23	0.79 ± 0.32	0.6–0.98
**CD45RO+ in CD4**	0.46 ± 0.26	0.34–0.58	0.57 ± 0.25	0.5–0.64	0.45 ± 0.13	0.36–0.64	0.47 ± 0.15	0.38–0.57
**CD45RO-CD26+ in CD4**	0.4 ± 0.24	0.29–0.51	0.56 ± 0.35	0.46–0.67	0.6 ± 0.31	0.38–0.82	0.51 ± 0.22	0.38–0.65
**CD45RO+CD26- in CD4**	0.13 ± 0.11	0.08–0.18	0.16 ± 0.11	0.13–0.2	0.09 ± 0.05**	0.06–0.13	0.14 ± 0.9	0.08–0.19
**CD45RO+CD26+ in CD4**	0.22 ± 0.11	0.18–0.27	0.3 ± 0.13*	0.26–0.34	0.25 ± 0.07	0.19–0.3	0.23 ± 0.08	0.24–0.29
**CD45RO+CD26++ in CD4**	0.11 ± 0.11	0.05–0.16	0.11 ± 0.06	0.09–0.13	O.11 ± 0.07	0.06–0.16	0.1 ± 0.1	0.04–0.13

n = number of samples; CI = confidence interval; SD = standard deviation; BT = biological therapy

*Values significantly different to No BT group at p < 0.05 (Student´s t-test)

** Values significantly different to anti-TNFα group at p < 0.05 (Student´s t-test)

# Values significantly different to control group (No BT) at p < 0.05 (Dunnett´s t-test)

These changes cannot be ascribed to disease parameters, which are only slightly worse in the anti-CD20 group (Table 1).

### CD4 subsets defined by CD26 levels correlate with the DAS28 index and other disease activity variables

**Fig 3:**
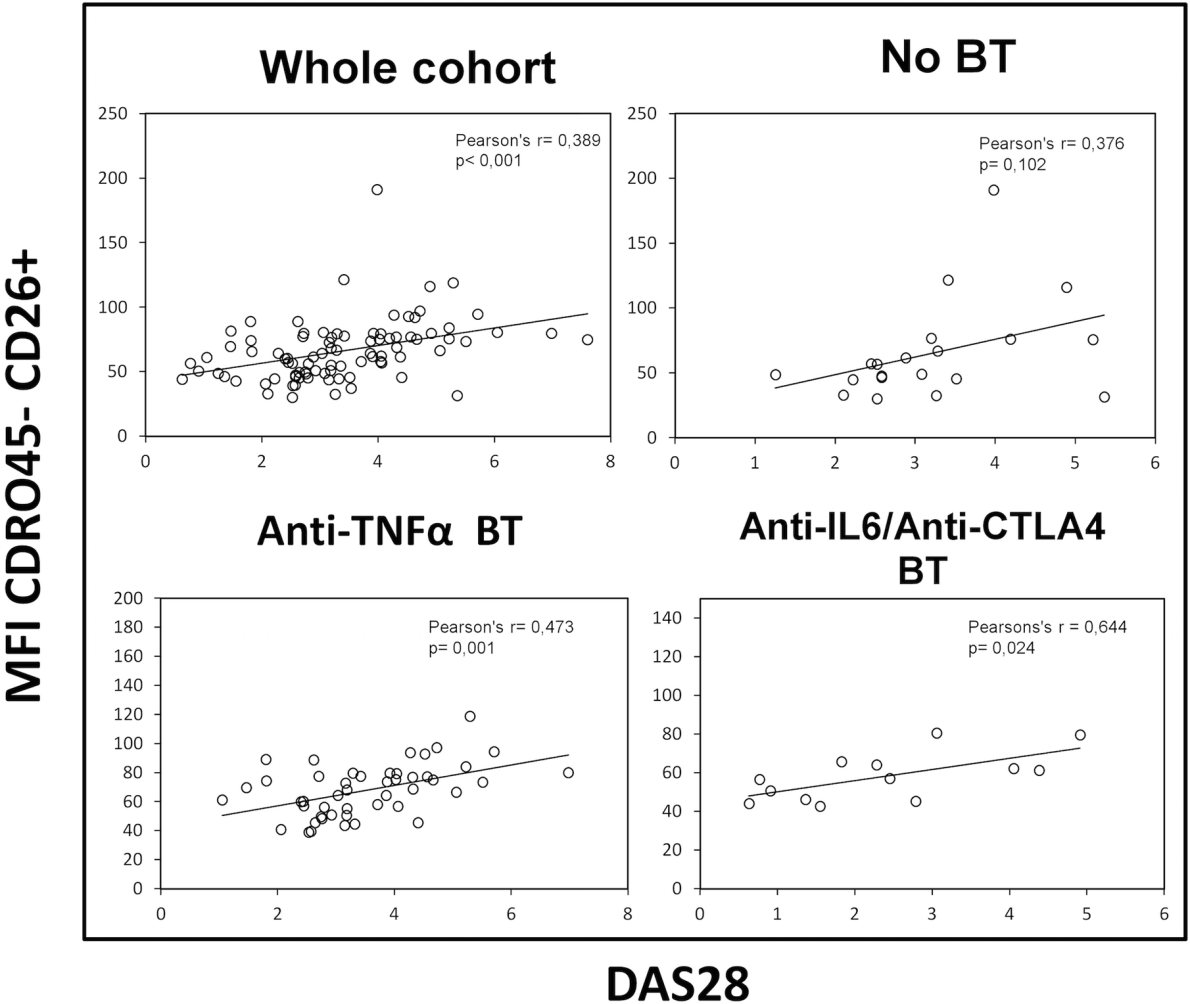
Correlation between disease activity and CD26^+^ naïve T cells. DAS28 (Disease Activity Score 28) correlations with CD26 cell surface density of circulating CD4 CD45R0^**-**^ CD26^**+**^ naïve T lymphocytes in the whole cohort of patients (upper left panel), in the No BT group (patients without biological activity, upper right panel), and in two groups of patients with biological therapies (lower panels). Pearson’s coefficient (r) and significance (p) data are insert in the panels.

For the RA patient cohort as a whole, statistically significant correlations between the DAS28 index and the frequencies (and total number of cells, data not shown) of several T cell subsets, as well as their surface CD26 levels, were observed. In particular, with the frequency of CD45R0^+^CD26^++^ (CD26high) population (r = 0.34, p = 0.001; Pearson) and their cell surface expression levels (MFI) of CD26 (r = 0.25, p = 0.017; Pearson), together with the CD45R0^-^CD26^+^ population (r = 0.38, p< 0.001; Pearson) (Fig 3). A similar analysis in each group of RA patients showed stronger (Fig 3) and additional correlations (data not shown). However, the CD45R0^+^CD26^-^ subset did not correlate with the DAS28 index.

**Fig 4:**
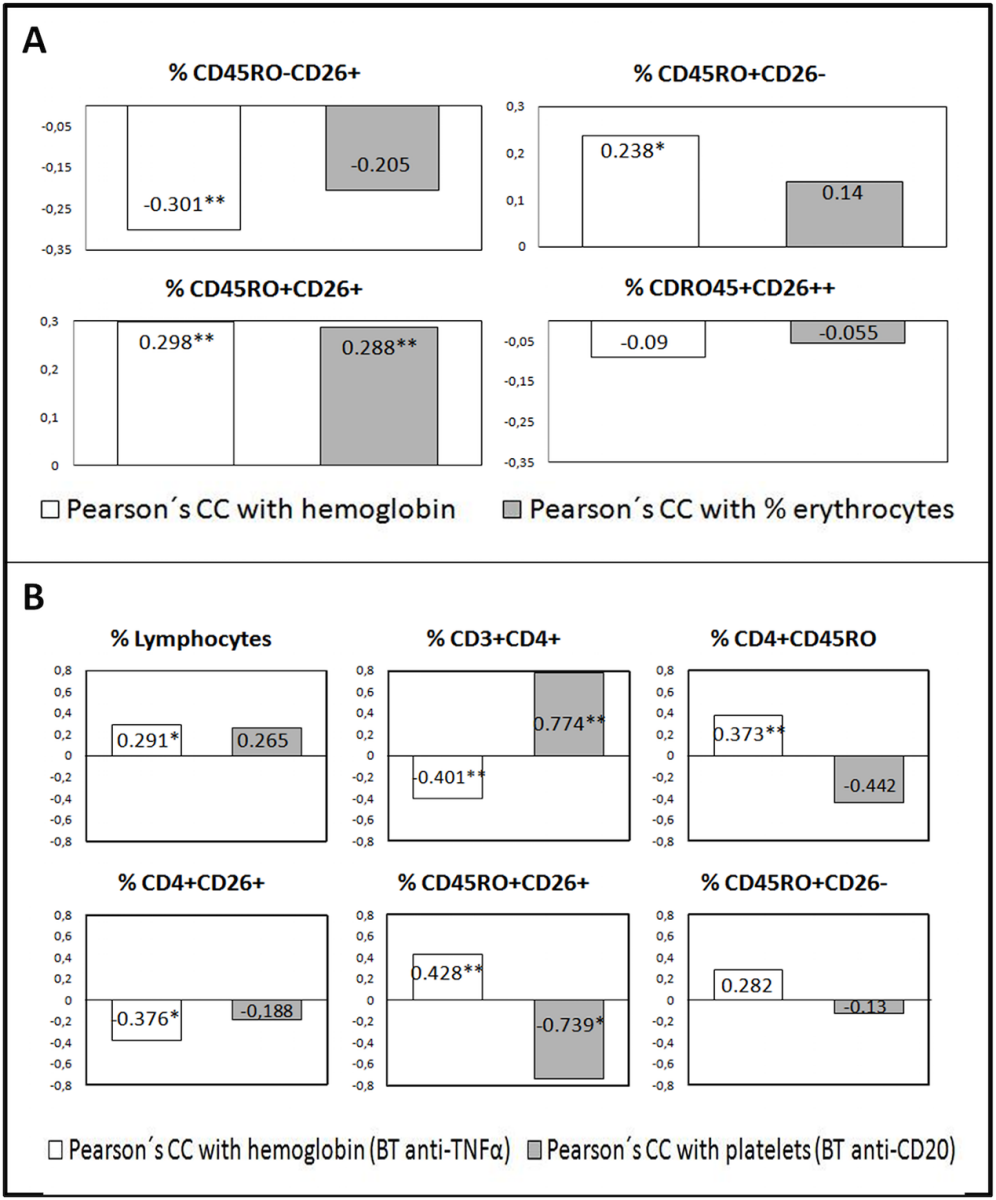
Correlations of CD4^+^ T cell subsets defined by surface CD45R0 and CD26 expression with non-DAS28 RA activity variables hemoglobin (Hb), hematocrit and the platelet count. A) In the whole cohort of patients a low positive correlations seen between the percentages of CD45R0^**+**^CD26^**+**^ and CD45R0^**+**^CD26^**-**^ subsets with Hb (white bars) (numbers represent Pearson’s coefficient, r, asterisks for statistical significance, *p<0.05, ** p<0.005) or % erythrocytes (dark bars), whereas the CD45R0^**-**^CD26^**+**^ subset showed a negative correlation and the CD26high subset did not correlate. B) Correlations of the four cell subsets in two patients’ groups. In the anti-TNF group some statistically significant correlations with Hb are shown for some T cell subsets (white bars). In the anti-CD20 group those cell populations show correlations with the platelet count (dark bars), generally in the opposite direction compared to the anti-TNF group. Non-significant correlations in the panels are shown only when the total cell number of the subset (instead of the percentage) was significant.

Additional results of correlations with the different components of the DAS28 index and with hemoglobin, hematocrit and the platelet count in each group of patients were found (data not shown). Many were different in each group of patients and could be linked to the physiological process of CD26 expression changes. To note, the CD45R0^+^CD26^-^ population did correlate with some of these non-DAS variables (Fig 4A, Fig 4B shows correlations for other subsets).

### Serum DPP-IV activity, sCD26 concentrations, and their correlations with the different CD4 subsets in the cohort of RA patients under different therapies

Serum DPP-IV activity was clearly lower in the RA patients when compared to healthy controls, however this reduction in enzyme activity did not correspond with a clear reduction in levels of sCD26 (Table 4). This reduction in DPP-IV activity is observed for all RA patients groups including the No BT group (milder cases) but a significant reduction in sCD26 is observed only for RA patients treated with anti-CD20 (Table 4). The anti-TNF group showed higher activity levels than the other groups, as expected [21], but still much lower than those from healthy donors. No correlation between serum DPP-IV activity or sCD26 levels and the DAS28 index or clinical and laboratory parameters for the different RA patients groups was found.

**Table 4:** Dipeptidyl peptidase 4 (DPP-IV) enzymatic activity levels and sCD26 concentration in serum of RA patients and healthy donors. Patients were additionally grouped according to the kind of therapy.

	DPP-IV activity (U L^-1^)			(sCD26) (g L^-1^)		
	n	Mean ± SD	CI (95%)	n	Mean ± SD	CI (95%)
**Healthy donors**	25	43.8 ± 16.74	36.61–50.42	25	0.26 ± 0.057	0.24–0.28
**RA patients**	106	30.28 ± 17.49*	26.91–33.65	107	0.23 ± 0.057*	0.22–0.24
**No BT**	21	28.50 ± 13.44**	22.38–34.62	22	0.22 ± 0.05	0.20–0.25
**Anti-TNFα BT**	58	33.06 ± 20.08**	27.78–38.34	58	0.24 ± 0.06	0.23–0.26
**Anti-CD20 BT**	12	27.04 ± 12.66**	18.99–35.09	12	0.20 ± 0.064**	0.17–0.24
**Anti-IL6R/Ig-CTLA4 BT**	15	24.63 ± 13.69**	17.05–32.21	15	0.24 ± 0.038	0.22–0.26

n = number of samples; CI = confidence interval; SD = standard deviation; RA = rheumatoid arthritis; BT = biological therapy

*Values significantly different to control group at p < 0.05 (Student´s t test)

**Values significantly different to control group at p < 0.05 (Dunnett´s t test)

There was a significant correlation between both variables (r = 0.55, p< 0.001; Pearson) in the whole cohort, however results were different in each group: No correlation in the No BT group, r = 0.59, p< 0.001 the anti-TNF group, r = 0.79, p = 0.002 the anti-CD20 group, and near significance r = 0.50, p = 0.060 the anti-IL6R/CTLA-4 group.

**Fig 5:**
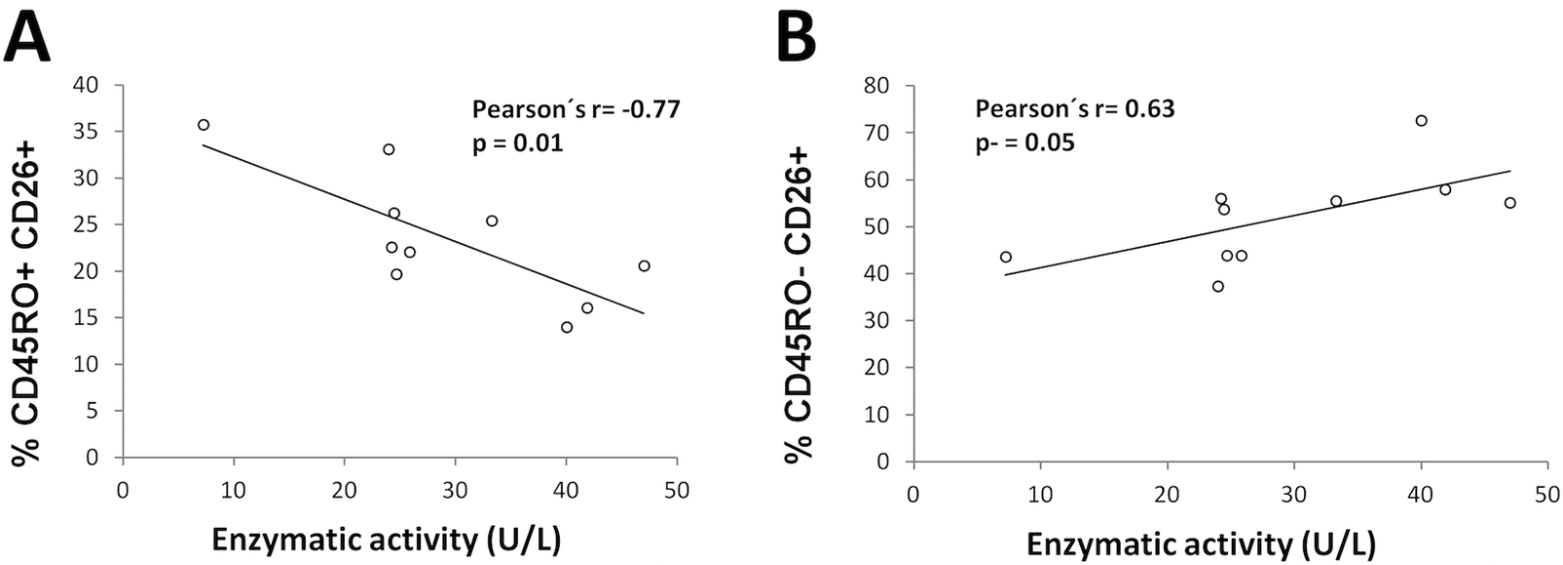
Serum dipeptidyl peptidase IV activity correlates with particular T cell populations in RA patients. As an example, only correlations for the anti-CD20 therapy group are shown. A) Negative correlation between percentages of effector/memory T cells (CD4^**+**^CD45R0^**+**^CD26^**+**^) and DPP-IV enzymatic activity. B) Positive correlation between percentages of naïve T cells (CD4^**+**^CD45R0^**-**^CD26^**+**^) and DPP-IV enzymatic activity. Pearson’s coefficient (r) and significance (p) data are insert in the panels.

Since the different therapies clearly affect serum DPP-IV activity and to a lesser extent sCD26 levels, we studied whether these changes correlate with any of the T cell population measured in these patients. In the whole cohort, a statistically significant correlation was found between the frequency (and the total number, data not shown) of lymphocytes and both DPP-IV activity and sCD26 levels (r = 0.45, p = 0.001). Importantly, levels of sCD26 correlated with the number of CD45R0^+^CD26^-^ cells, the population with the highest reduction seen in RA patients, as well as negatively with cell surface CD26 MFI in the CD45R0^-^CD26^+^ population. In each of the different groups of patients, significant correlations were also observed for additional subsets (Fig 5 with the anti-CD20 group as example). However, no relation was found with the CD26high (CD26^++^) population.

### Th17 and Th22 cell subsets in RA

Th17 and Th22 pro-inflammatory cells were measured in order to analyse whether the different therapies affected their distribution since it has been previously reported that CD45R0^+^ Th17 are CD26high [21] and Th22 cells are also CD45R0^+^.

**Fig 6:**
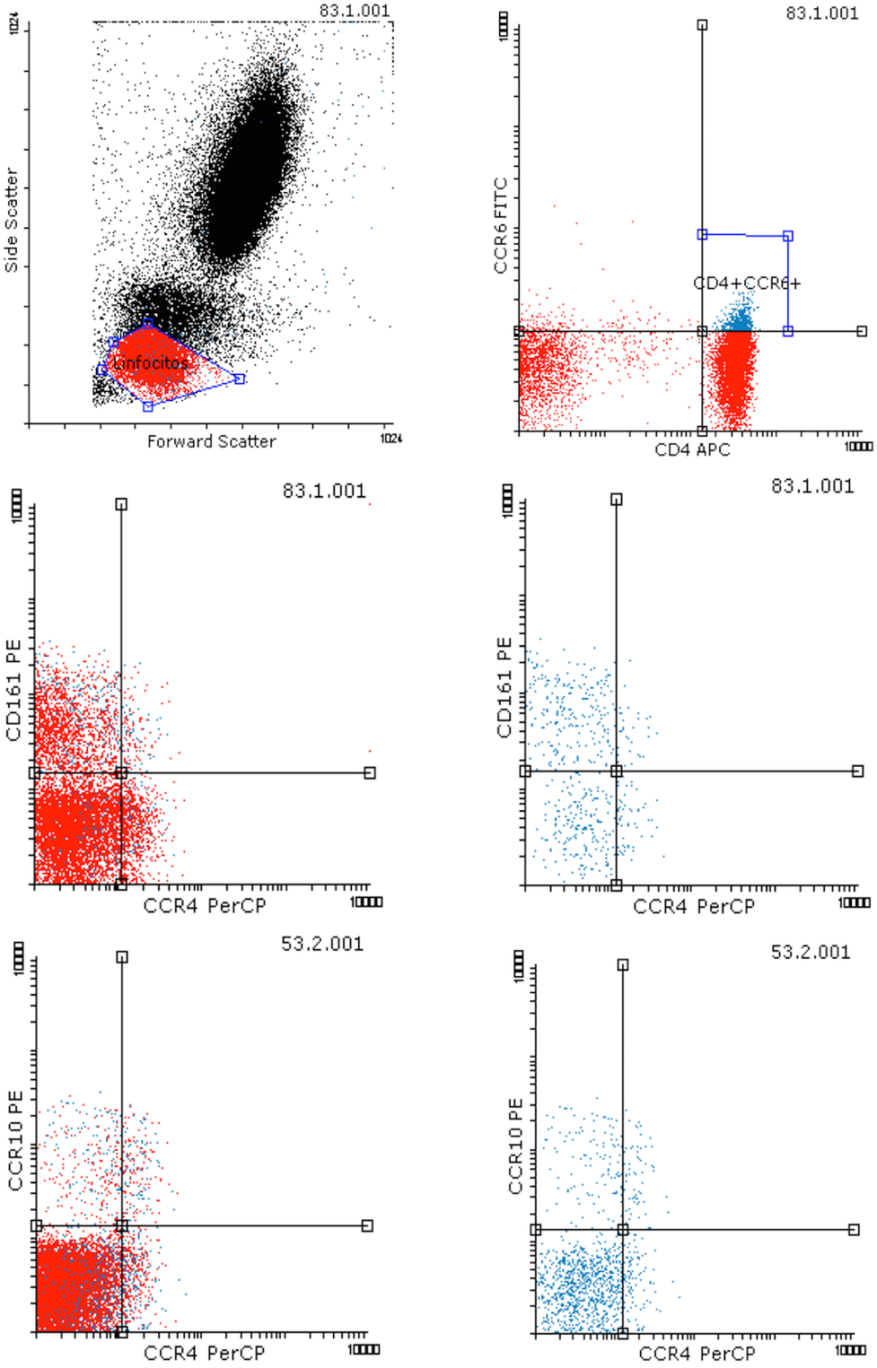
T helper 17 and 22 cell subsets defined by surface CD4, CCR6 (CD196), CD161 and CCR4 (CD194). A) Representative flow cytometry dot-plot showing lymphocyte gating strategy. B) Representative dot-plot showing that CCR6 is expressed mostly by CD4^**+**^ T lymphocytes. C) and D) Dot-plots showing the differential expression of CD161 and CCR4 in the whole lymphocyte region gated in A (C) or in the CD4^**+**^CCR6^**+**^ region gated in B (D). The upper left and right subsets of CD4^**+**^CCR6^**+**^CD161^**+**^, CCR4^**-**^ and CCR4^**+**^ respectively, were recorded for each patient as different Th17 subsets. E) and F) Dot-plots showing the differential expression of CCR10 and CCR4 in the whole lymphocyte region gated in A (C) or in the CD4^**+**^CCR6^**+**^ region gated in B (D). The upper left and right subsets of CD4^**+**^CCR6^**+**^CCR10^**+**^, CCR4^**-**^ and CCR4^**+**^ respectively, were recorded for each patient as different Th22 subsets.

Using surface markers (see Methods and Fig 6), a 0.65 ± 0.06% of the CD4 T cells were canonical Th17 (CD4^+^CCR6^+^CD161^+^) and 0.1 ± 0.05% were Th22 (CD4^+^CCR6^+^CCR10^+^) in our cohort of RA patients (Tables 5 and 6). These populations were studied for the first time in RA as split CCR4+ or CCR4- Th17 and Th22 subsets [30]. Correlations found between these as-defined populations confirmed the specificity of the subsets (data not shown). Interestingly, the Th17 CD4^+^CCR6^+^CD161^+^CCR4^-^ population was higher compared to the CCR4^+^ one, and the ratio between them quite variable among the different therapy groups (from 2.5 to 8-fold), a variation due to changes in the frequency (and number) of the CCR4^+^ cells (Table 5). In contrast among the Th22 cells, both CCR4 populations were quite similar in the No BT group, showing a dramatic increase the CCR4^-^ cells in the biological therapy groups (Table 6). Correlations with variables of RA disease confirmed the relationship of these four Th17 and Th22 subsets with RA (data not shown); however, no correlations with the DAS28 index were found.

**Table 5:** Percentages and total number of different CD4 T subsets gated according to the CCR6, CCR4 and CD161 staining. Patients were grouped according to the kind of therapy.

	No BT (n = 10)		Anti-TNFα BT (n = 34)		Anti-CD20 BT (n = 9)		Anti-IL6R/Ig-CTLA4 BT (n = 12)	
	Mean ± SD	CI (95%)	Mean ± SD	CI (95%)	Mean ± SD	CI (95%)	Mean ± SD	CI (95%)
**% lymphocytes**	27.75 ± 7.33*	22.50–32.99	27.6 ± 10.69* #	23.87–31.33	20.28 ± 4.69* #	16.68–23.88	20.66 ± 11.29	13.48–27.83
**% lymphocytes CD4** ^**+**^ **CCR6** ^**+**^	1.38 ± 0.98	0.68–2.08	1.54 ± 0.94	1.21–1.87	1.54 ± 1.07	0.72–2.37	1.03 ± 0.64	0.63–1.44
**% CD161** ^**+**^ **CCR4** ^**+**^ **in CD4** ^**+**^ **CCR6** ^**+**^	15.15 ± 12.11*	5.84–24.47	6.76 ± 5.39	4.82–8.7	10.18 ± 6.02*	5.15–15.21	5.78 ± 2.85*	3.86–7.7
**% CD161** ^**+**^ **CCR4** ^**-**^ **in CD4** ^**+**^ **CCR6** ^**+**^	39.25 ± 8.32	32.85–45.64	44.21 ± 11.8	39.96–48.47	43.02 ± 12.71	32.4–53.64	41.95 ± 8.82	36.02–47.87
**Number of lymphocytes** (**x 10** ^**9**^ **cel L** ^**-1**^ **)**	2.12 ± 0.94	1.7–2.53	2.73 ± 0.98	2.47–2.98	2.07 ± 0.8	1.6–2.53	2.32 ± 0.54	2.01–2.62
**Number of lymphocytes CD4** ^**+**^ **CCR6** ^**+**^ (**x 10** ^**9**^ **cel L** ^**-1**^ **)**	0.12 ± 0.11	0.041–0.21	0.16 ± 0.11	0.12–0.2	0.15 ± 0.13	0.05–0.25	0.12 ± 0.05	0.09–0.15
**Number of CD161** ^**+**^ **CCR4** ^**+**^ **in CD4** ^**+**^ **CCR6** ^**+**^ (**x 10** ^**6**^ **cel L** ^**-1**^ **)**	16.84 ± 14.61	5.62–28.07	9.54 ± 10.76	5.66–13.42	9.63 ± 62.77	4.39–14.88	6.45 ± 3.78	3.91–8.9
**Number of CD161** ^**+**^ **CCR4** ^**-**^ **in CD4** ^**+**^ **CCR6** ^**+**^ (**x 10** ^**6**^ **cel L** ^**-1**^ **)**	45.71 ± 51.93	5.79–85.63	61.73 ± 40.37	47.18–76.29	46.67 ± 30.83	20.89–72.44	51.66 ± 33.4	29.22–74.1

n = number of samples; CI = confidence interval; SD = standard deviation; BT = biological therapy

* Values significantly different among groups (not specified) at p < 0.05 (Student´s t test)

# Values significantly different among groups at p < 0.05 (Dunnett´s C test)

**Table 6:** Percentages and total number of different CD4 T subsets gated according to the CCR6, CCR4 and CCR10 staining. Patients were grouped according to the kind of therapy.

	No BT (n = 4)		Anti-TNFα BT (n = 33)		Anti-CD20 BT (n = 5)		Anti-IL6R/Ig-CTLA4 BT (n = 7)	
	Mean ± SD	CI (95%)	Mean ± SD	CI (95%)	Mean ± SD	CI (95%)	Mean ± SD	CI (95%)
**% lymphocytes**	24.98 ± 5.08*	16.89–33.06	27.76 ± 10.52* #	24.02–31.49	19.29 ± 6.2	11.6–26.98	14.43 ± 6.8* #	8.14–20.71
**% lymphocytes CD4** ^**+**^ **CCR6** ^**+**^	1.56 ± 0.87	0.16–2.95	2.21 ± 1.29*	1.76–2.67	1.93 ± 1.61	(-)0.06–3.93	0.85 ± 0.38*	0.5–1.2
**% CCR10** ^**+**^ **CCR4** ^**+**^ **in CD4** ^**+**^ **CCR6** ^**+**^	3.15 ± 1.68	(-)1.01–7.31	4.96 ± 4.39	3.35–6.57	4.94 ± 1.14	3.12–6.77	4.81 ± 1.53	3.2–6.41
**% CCR10** ^**+**^ **CCR4** ^**-**^ **in CD4** ^**+**^ **CCR6** ^**+**^	3.77 ± 3.11* ¢	(-)3.95–11.49	9.5 ± 12.18¢	5.03–13.97	10.62 ± 3.53*	5–16.25	22.57 ± 18.76	2.87–42.26
**Number of lymphocytes** (**x 10** ^**9**^ **cel L** ^**-1**^ **)**	2.12 ± 0.94	1.7–2.53	2.73 ± 0.98	2.47–2.98	2.07 ± 0.8	1.6–2.53	2.32 ± 0.54	2.01–2.62
**Number of lymphocytes CD4** ^**+**^ **CCR6** ^**+**^ (**x 10** ^**9**^ **cel L** ^**-1**^ **)**	0.16 ± 0.14	(-)0.07–0.38	0.23 ± 0.15*	0.18–0.28	0.2 ± 0.17	(-)0.01–0.40	0.15 ± 0.05*	0.11–0.2
**Number of CCR10** ^**+**^ **CCR4** ^**+**^ **in CD4** ^**+**^ **CCR6** ^**+**^ (**x 10** ^**6**^ **cel L** ^**-1**^ **)**	2.48 ± 1.64*	(-)1.59–6.55	10.82 ± 13.5	5.87–15.78	5.95 ± 2.03	2.71–9.19	6.73 ± 2.48*	4.12–9.33
**Number of CCR10** ^**+**^ **CCR4** ^**-**^ **in CD4** ^**+**^ **CCR6** ^**+**^ (**x 10** ^**6**^ **cel L** ^**-1**^ **)**	2.14 ± 0.69*	0.42–0.3.8	18.34 ± 17.85	11.79–24.88	12.97 ± 4.99*	5.02–20.92	32.73 ± 27.81*	3.55–61.92

n = number of samples; CI = confidence interval; SD = standard deviation; BT = biological therapy

* Values significantly different among groups (not specified) at p < 0.05 (Student´s t test)

# Values significantly different among groups at p < 0.05 (Scheffé´s test)

¢ Values significantly different among groups at p < 0.05 (Dunnett´s C test)

### Correlations among Th17 and Th22 cells and the different CD4 subsets and serum DPP-IV/CD26

**Fig 7:**
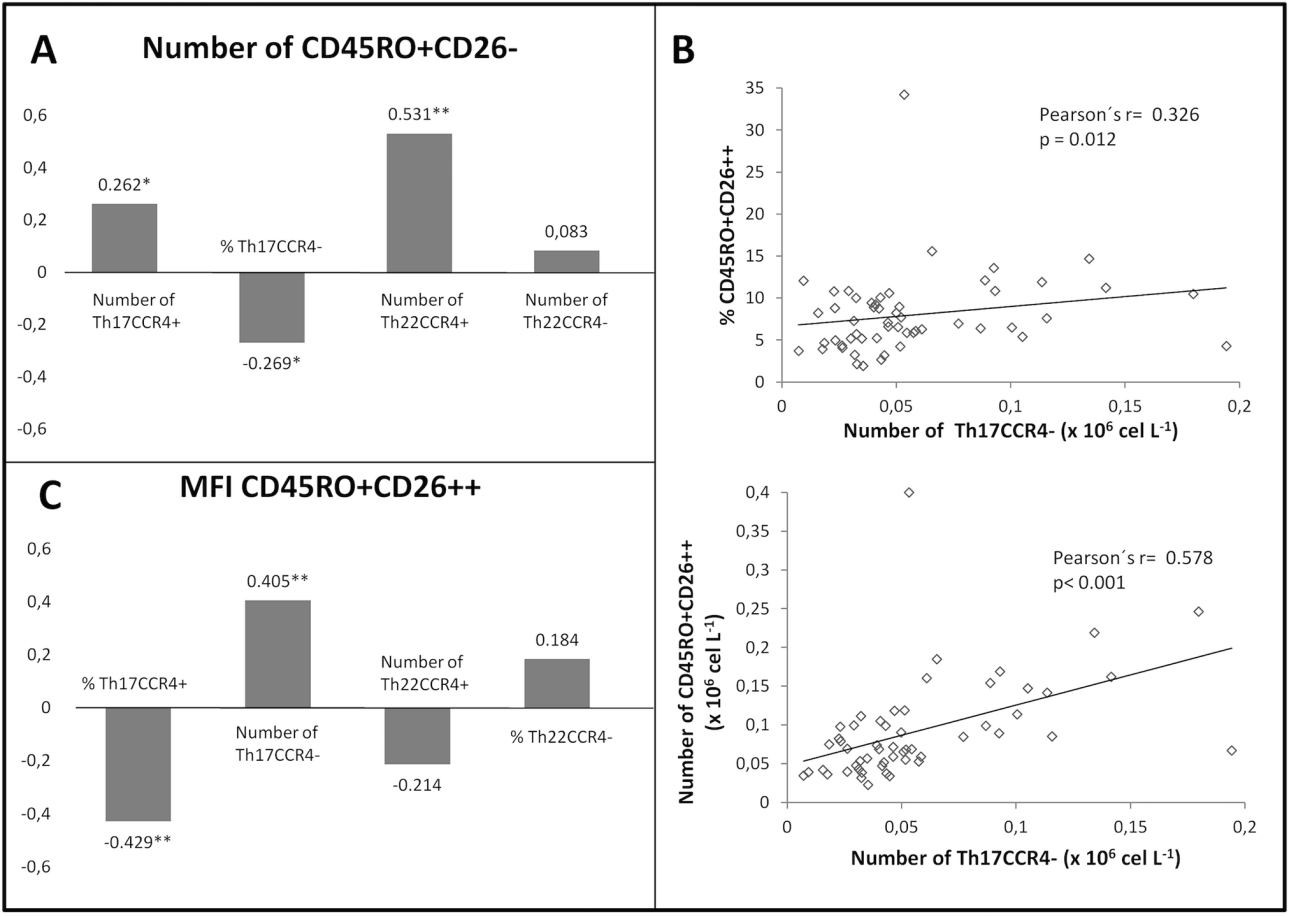
Correlation between Th17 and Th22 and CD26 cell subsets. A) In the whole cohort, the frequency of CD45R0^**+**^CD26^**-**^ subset correlates negatively with the Th17 CCR4^**-**^ cells but positively with the other Th17 and Th22 subsets (Th22CCR4^**-**^ did not correlate in the whole cohort but it did among different patients’ groups, data not shown). B) The number of Th17CCR4^**-**^ cells correlates with the percentage of CD26high cells (upper graph) and strongly with absolute cell number (lower graph). C) The cell surface CD26 (MFI) in the CD26high population positively correlates with the number of Th17CCR4^**-**^ cells and negatively with both the Th17CCR4^**+**^ and Th22CCR4^**+**^ cells (Th22CCR4^**+**^ did not correlate in the whole cohort but it did among different patients’ groups, data not shown). Numbers represent Pearson’s coefficient, r, (*p<0.05, ** p<0.005).

Although CD26 expression levels in different CD4 T cell subsets cannot be ascribed to either Th17 or Th22 cell populations, an important finding contributing to the knowledge of these populations was that only the Th17CCR4^-^, but not the Th17CCR4^+^ cell subset, is CD26^++^, similar to Th1 cells (Fig 7, and flow cytometry data not shown).

Correlations of Th17CCR4^+^ and Th22CCR4^+^ cells (and weakly, Th22CCR4^-^ cells) with the CD45R0^+^CD26^+^ and CD45R0^+^CD26^-^ subsets (data not shown) point to a mix of CD26^+^ and−cells populations in these subsets.

Correlations between CD26 subsets and Th17 or Th22 populations also changed under the different therapies. The Th22CCR4^-^ cells (percentage and absolute numbers) correlated very strongly (r = 0.91, p = 0.012, the latter) with the CD26^++^ only in the anti-IL6R/CTLA4 group but not in the others. To note, in addition, the strong correlation between the Th17CCR4^+^ cells with the CD45R0^+^CD26^-^ subset (the one non affected by therapies) in the anti-TNF and anti-CD20 groups (r = 0.89, p = 0.017 for the latter); or the Th17CCR4^-^ cells with the same CD45R0^+^CD26^-^ subset (r = 0.86, p = 0.003) in the No BT, pointing to a regulation of CD26 expression in these T cells.

The relationship of serum and lymphocyte CD26 is further supported by the fact that the Th22 CCR4^+^ subset correlated positively with sCD26/DPP-IV levels (r = 0.85, p = 0.032 for the activity), mainly in the anti-IL6R/CTLA4 group where more cells of that subset were found (Table 5).

## Discussion

Although DPP-IV activity levels are decreased both in serum and synovial fluid of RA patients [11,4,6–10] and this decrease is known to be associated to disease activity [8,10], little is known about the events leading to this decrease, apart from the fact that RA patients display higher percentages of CD4^+^CD26^+^ T cells and CD26 cell surface density [15,18], an opposite situation when compared to SLE patients [6]. In the present work, quantification of DPP-IV enzymatic activity and sCD26 in serum as well as quantification of CD26 on the cell surface of different CD4 T cell subsets has been studied in RA patients undergoing different treatments.

The slightly higher percentages of experienced T cells (CD45R0^+^) as well as CD4^+^CD26^+^ cells found in non-treated RA patients [15,18] are similar to those found in patients under therapy for at least a year. Since CD26 has been defined as an activation marker for T cells, the common interpretation for this observation is that the inflammatory process leads to an up-regulation of the CD26 expression [11,15,18]. Alternatively, this could be explained by an increment in the CD45R0^+^CD26^++^ cell (CD26high) subset, mainly pro-inflammatory effector Th1 lymphocytes [20,24,25]. In fact, this CD26^++^ subset correlates with the clinical severity of another autoimmune disease, multiple sclerosis [20,34]. We found, however, that the major difference in RA patients was the decrease of the CD4^+^CD45R0^+^CD26^-^ population, which can explain the increment of CD4^+^CD26^+^ cells on their own. For example, the anti-CD20 group showed the higher diminution in the frequency of these cells accompanied with the higher percentage of CD26^+^ cells.

In fact, none of the therapies lead to the recovery of normal numbers of CD45R0^+^CD26^-^ cells. However, therapies were recovering other subsets; for example, while the No BT group, a group similar to RA patients without therapy [18] showed the highest percentages of CD26^++^ cells, a normalized frequency of CD26^++^ cells was found in the anti-IL6R/Ig-CTLA4 subset. Equally, examining the density of CD26 on the T cell surface, it shows that the effect of each therapy was different.

Reduction in CD45^+^CD26^-^ cells is a new pathway to be investigated in the early diagnosis of RA since it is a very complex population that includes central memory T cells (Tcm), regulatory T cells (Tregs) and others [20]. Although, baseline levels of the CD45R0^+^CD26^-^ subset were not recovered with any of the therapies at the time of sampling, a correlation with hemoglobin levels was observed in the anti-IL-6R group. IL-6, together with TNF-**α** [35], is an inflammatory cytokine critically involved in chronic disease´ anemia, the major reason for the low hemoglobin levels in RA patients. In fact, it has been recently reported that hemoglobin levels increase after biologic therapies, mainly with anti-IL-6R [35], indicating that anti-IL6R therapy could lead to the recovery of this population.

The importance of the CD26high^(++)^ subset, mostly Th1, in RA is highlighted by the strong correlation (similar to CRP, data not shown) between both the frequency and CD26 surface density with DAS28, particularly in the No BT group of patients. However, from a diagnostic point of view, this CD26high population also correlates with severity score in other autoimmune diseases such as multiple sclerosis [20, 34]. It is important to note that biological therapy in RA, which is not used in MS patients, (mainly anti-IL6R, Ig-CTLA4 and anti-CD20, but not anti-TNF) modulates this population. However, the CD26^++^ subset was not the only one, the R0^-^CD26^+^ subset correlated stronger with DAS28.

Two possible mechanisms of action of the different therapies on the Th1 subset can be inferred from this data: a) a down-regulation of CD26 in the Th1 subset (more R0^+^CD26^+^, intermediate, cells), and/or b) blocking of an early event of the T cell activation or, alternatively, a loss of the CD45R0 isoform (more R0^-/low^ CD26^+^ cells), as it has been previously described for some central or effector memory populations [36]. The first mechanism showed stronger correlation with clinical data such as PGA, ESR and platelet count whereas the second correlated with joint damage, CRP and negatively correlated with ESR or hemoglobin. These are different features of the inflammatory process [23,27,28,35,37] hence it can be inferred that these data are physiologically relevant.

The results of Th17/Th22 cells are important. First, the methodological approach using several markers to define these lineages [21, 22, 26–28] may be a better reflection of the in vivo situation than the ex vivo approach [20, 21, 26–28]. Secondly, recently two Th17 subsets, have been described, the standard CD4^+^CCR6^+^CD161^+^CCR4^+^ and the new Th17CCR4^-^ cells [30]. We also analysed the Th22 subset in RA using a similar approach since a Th22CCR4^-^ population was suspected [38]. We show for the first time the involvement of these four subsets in RA.

We found a much higher Th17/Th22 ratio, in contrast t to previous results [21], even when only the CCR4^+^ populations were studied (ratio = 4). Canonical Th17 and Th22 populations did not correlate with DAS28, contrary to reported data [22]. This discrepancy could be related to either the larger cohort studied in the present study, to the more aggressive disease in patients described in that study (DAS28 = 5.2), or the fact that these patients did not receive immunosuppressive or immunomodulatory drugs for at least 2 months prior to sampling compared to patients in the present work. If this is the case, then the CD26 subsets are more related to RA activity, since they still correlated with DAS in the same conditions. Th17 and Th22 cell populations were related to RA in the present work mainly because all biological therapies, in particular anti-IL-6R/Ig-CTLA4, changed their number and ratio.

Interestingly, the Th17CCR4^-^ subset but not the TGF-**β**-secreting Th17CCR4^+^ [30], is included in the CD26^++^ population together with Th1 cells [21]. TGF-**β** can down-regulate CD26/DPPIV expression on T cells, mainly on Th1 [39], suggesting an autocrine loop acting on the Th17CCR4^+^ subset. The same authors showed that IL-6 has no effect on CD26 expression [39] suggesting indirect effects of anti-ILR therapy. On the other hand, the Th22CCR4^-^ cells seemed mostly CD26^-^ whereas the Th17CCR4^+^ and Th22CCR4^+^ cells are a mix of CD26^+^ and CD26^–^ cells. To note, the increase of Th17CCR4^-^ CD26^++^ cells by biological therapies suggest that not all CD26^++^ cells are related to the inflammatory pathway of Th1 cells.

We also investigated the behaviour of serum sCD26 and DPP-IV [4,5,8,9]. Their levels were very low, particularly for DPP-IV enzymatic activity, in the No BT group. Both biomarkers significantly correlated in the whole cohort, however they did not correlate in the No BT group, while in the anti-CD20 group the correlation was quite strong. Similarly, while in the anti-TNF group sCD26 levels recovered, confirming previous reports [19], the enzymatic activity did not, and anti-CD20 partially restored the activity but not the sCD26 levels. These results point to a factor distinct to the protein concentration but related to the enzymatic activity in RA. This has been previously shown for other diseases [11,40] and attempts to explain it have been reported [8,9,40]. In addition, correlation data confirmed that a very important fraction of the soluble CD26 originates from lymphocytes, supporting previous hypotheses [11]. Since DPP-IV has been implicated in chemotaxis [15,41], these data open new clues to study its role in RA or other diseases’ biological history.

## Conclusion

The CD26 expression levels on several T helper populations can provide clues to identify biomarkers for earlier stages of RA. Also, independent CCR4^+^ and CCR4^-^ Th17 and Th22 subsets were identified in RA. All these subsets were affected in distinct ways by therapies. A follow-up of the evolution of these parameters in a further longitudinal study will give us more data.

## Supporting Information

Due to patient privacy concerns, data may be requested from the corresponding author.

## References

[1] Aletaha D, Neogi T, Silman AJ, Funovits J, Felson DT, Bingham CO III, et al Rheumatoid arthritis classification criteria: an American College of Rheumatology/European League Against Rheumatism collaborative initiative. Arthritis Rheum 2010;62:2569–81. 10.1002/art.27584 20872595

[2] Gerlag DM, Raza K, van Baarsen LG, Brouwer E, Buckley CD, Burmester GR, et al EULAR recommendations for terminology and research in individuals at risk of rheumatoid arthritis: report from the Study Group for Risk Factors for Rheumatoid Arthritis. Ann Rheum Dis 2012;71:638–41. 10.1136/annrheumdis-2011-200990 22387728 PMC3329228

[3] Biswas S, Sharma S, Saroha A, Bhakuni DS, Malhotra R, Zahur M, et al Identification of novel autoantigen in the synovial fluid of rheumatoid arthritis patients using an immunoproteomics approach. PLoS ONE 2013;8: e56246 10.1371/journal.pone.0056246 23418544 PMC3572018

[4] Hagihara M, Ohhashi M, Nagatsu T. Activities of dipeptidyl peptidase II and dipeptidyl peptidase IV in mice with lupus erythematosus-like syndrome and in patients with lupus erythematosus and rheumatoid arthritis. Clin Chem 1987;33:1463–5. 2886236

[5] Gotoh H, Hagihara M, Nagatsu T, Iwata H, Miura T. Activities of dipeptidyl peptidase ii and dipeptidyl peptidase iv in synovial fluid from patients with rheumatoid arthritis and osteoarthritis. Clin Chem 1989;35: 1016–8. 2567214

[6] Cordero OJ, Salgado FJ, Mera-Varela A, Nogueira M. Interleukin 12, interleukin 15, soluble CD26 and adenosine deaminase levels in the sera of rheumatoid arthritis patients. Rheumatol Int 2001;21: 69–74. 11732862 10.1007/s002960100134

[7] Sromova L, Mareckova H, Sedova L, Balaziova E, Sedo A. Dipeptidyl peptidase-IV in synovial fluid and in synovial fluid mononuclear cells of patients with rheumatoid arthritis. Clin Chim Acta 2010;411:1046–50. 10.1016/j.cca.2010.03.034 20361950

[8] Kobayashi H, Hosono O, Mimori T, Kawasaki H, Dang NH, Tanaka H, et al Reduction of serum soluble CD26/dipeptidyl peptidase IV enzyme activity and its correlation with disease activity in systemic lupus erythematosus. J Rheumatol 2002;29: 1858–66. 12233879

[9] Cuchacovich M, Gatica H, Pizzo SV, Gonzalez-Gronow M. Characterization of human serum dipeptidyl peptidase IV (CD26) and analysis of its autoantibodies in patients with rheumatoid arthritis and other autoimmune diseases. Clin Exp Rheumatol 2001;19: 673–80. 11791639

[10] Stancíková M, Lojda Z, Lukác J, Ruzicková M. Dipeptidyl peptidase IV in patients with systemic lupus erythematosus. Clin Exp Rheumatol 1992;10: 381–5. 1356680

[11] Cordero OJ, Salgado FJ, Nogueira M. On the origin of serum CD26 and its altered concentration in cancer patients. Cancer Immunol Immunother 2009;58: 1723–47. 10.1007/s00262-009-0728-1 19557413 PMC11031058

[12] Proost P, Mahieu F, Schutyser E, Van Damme J. Posttranslational processing of chemokines. Methods Mol Biol 2004;239:27–44. 14573907 10.1385/1-59259-435-2:27

[13] Narducci MG, Scala E, Bresin A, Caprini E, Picchio MC, Remotti D, et al Skin homing of Sezary cells involves SDF-1-CXCR4 signaling and down-regulation of CD26/dipeptidylpeptidase IV. Blood 2006;107: 1108–15. 16204308 10.1182/blood-2005-04-1492

[14] Buljevic S, Detel D, Pucar LB, Mihelic R, Madarevic T, Sestan B, et al Levels of dipeptidyl peptidase IV/CD26 substrates neuropeptide Y and vasoactive intestinal peptide in rheumatoid arthritis patients. Rheumatol Int. 2013;33:2867–74. 10.1007/s00296-013-2823-z 23864142

[15] Muscat C, Bertotto A, Agea E, Bistoni O, Ercolani R, Tognellini R, et al Expression and functional role of 1F7 (CD26) antigen on peripheral blood and synovial fluid T cells in rheumatoid arthritis patients. Clin Exp Immunol 1994;98: 252–6. 7955530 10.1111/j.1365-2249.1994.tb06134.x PMC1534402

[16] Havre PA, Dang LH, Ohnuma K, Iwata S, Morimoto C, Dang NH. CD26 expression on T-anaplastic large cell lymphoma (ALCL) line Karpas 299 is associated with increased expression of versican and MT1-MMP and enhanced adhesion. BMC Cancer 2013;13:517 10.1186/1471-2407-13-517 24180670 PMC4228418

[17] Havre PA, Abe M, Urasaki Y, Ohnuma K, Morimoto C, Dang NH. CD26 expression on T cell lines increases SDF-1-α-mediated invasion. Br J Cancer 2009;101:983–91. 10.1038/sj.bjc.6605236 19654580 PMC2743358

[18] Ellingsen T, Hornung N, Møller BK, Hjelm-Poulsen J, Stengaard-Pedersen K. In active chronic rheumatoid arthritis, dipeptidyl peptidase IV density is increased on monocytes and CD4(+) T lymphocytes. Scand J Immunol 2007; 66: 451–7. 17850590 10.1111/j.1365-3083.2007.01966.x

[19] Mavropoulos JC, Cuchacovich M, Llanos C, Aguillón JC, Gatica H, Pizzo SV, et al Anti-tumor necrosis factor-α therapy augments dipeptidyl peptidase IV activity and decreases autoantibodies to GRP78/BIP and phosphoglucose isomerase in patients with rheumatoid arthritis. J Rheumatol 2005;32: 2116–24. 16265688

[20] Krakauer M, Sorensen PS, Sellebjerg F. CD4(+) memory T cells with high CD26 surface expression are enriched for Th1 markers and correlate with clinical severity of multiple sclerosis. J Neuroimmunol 2006;181:157–64. 17081623 10.1016/j.jneuroim.2006.09.006

[21] Bengsch B, Seigel B, Flecken T, Wolanski J, Blum HE, Thimme R. Human Th17 cells express high levels of enzymatically active dipeptidylpeptidase IV (CD26). J Immunol 2012;188:5438–47. 10.4049/jimmunol.1103801 22539793

[22] Zhang L, Li Y-g, Li Y-h, Qi L, Liu XG, Yuan CZ, et al Increased frequencies of Th22 cells as well as Th17 cells in the peripheral blood of patients with ankylosing spondylitis and rheumatoid arthritis. PLoS ONE 2012;7: e31000 10.1371/journal.pone.0031000 22485125 PMC3317658

[23] Wolfe F, Kleinheksel SM, Cathey MA, Hawley DJ, Spitz PW, Fries JF. The clinical value of the Stanford Health Assessment Questionnaire Functional Disability Index in patients with rheumatoid arthritis. J Rheumatol 1988;15:1480–8. 3204597

[24] Salgado FJ, Lojo J, Alonso-Lebrero JL, Lluis C, Franco R, Cordero OJ, et al A role for interleukin-12 in the regulation of T cell plasma membrane compartmentation. J Biol Chem 2003;278: 24849–57. 12676959 10.1074/jbc.M212978200

[25] Ibegbu CC, Xu YX, Fillos D, Radziewicz H, Grakoui A, Kourtis AP. Differential expression of CD26 on virus-specific CD8(+) T cells during active, latent and resolved infection. Immunology 2009;126:346–53. 10.1111/j.1365-2567.2008.02899.x 18657205 PMC2669815

[26] Wan Q, Kozhaya L, ElHed A, Ramesh R, Carlson TJ, Djuretic IM, et al Cytokine signals through PI-3 kinase pathway modulate Th17 cytokine production by CCR6+ human memory T cells. J Exp Med 2011;208:1875–87. 10.1084/jem.20102516 21825017 PMC3171088

[27] Eyerich S, Eyerich K, Pennino D, Carbone T, Nasorri F, Pallotta S, et al Th22 cells represent a distinct human T cell subset involved in epidermal immunity and remodeling. J Clin Invest 2009;119:3573–85. 10.1172/JCI40202 19920355 PMC2786807

[28] Truchetet ME, Brembilla NC, Montanari E, Allanore Y, Chizzolini C. Increased frequency of circulating Th22 in addition to Th17 and Th2 lymphocytes in systemic sclerosis: association with interstitial lung disease. Arthritis Res Ther 2011;13:R166 10.1186/ar3486 21996293 PMC3308100

[29] Annunziato F, Romagnani S. Do studies in humans better depict Th17 cells? Blood 2009;114:2213–9. 10.1182/blood-2009-03-209189 19494349

[30] Zhao F, Hoechst B, Gamrekelashvili J, Ormandy LA, Voigtländer T, Wedemeyer H, et al Human CCR4+ CCR6+ Th17 cells suppress autologous CD8+ T cell responses. J Immunol 2012;188:6055–62. 10.4049/jimmunol.1102918 22615204 PMC3370143

[31] Cordero OJ, Salgado FJ, Viñuela JE, Nogueira M. Interleukin-12 enhances CD26 expression and dipeptidyl peptidase IV function on human activated lymphocytes. Immunobiology 1997;197:522–33. 9413751 10.1016/s0171-2985(97)80084-8

[32] De Chiara L, Rodríguez-Piñeiro AM, Cordero OJ, Rodríguez-Berrocal FJ, Ayude D, Rivas-Hervada, et al Soluble CD26 levels and its association to epidemiologic parameters in a sample population. Dis Markers 2009;27:311–6. 10.3233/DMA-2009-0679 20075514 PMC3835055

[33] Lambeir AM, Durinx C, Scharpé S, De Meester I. Dipeptidyl-peptidase IV from bench to bedside: an update on structural properties, functions, and clinical aspects of the enzyme DPP IV. Crit Rev Clin Lab Sci 2003;40: 209–94. 12892317 10.1080/713609354

[34] Tejera-Alhambra M, Casrouge A, de Andrés C, Ramos-Medina R, Alonso B, Vega J, et al Low DPP4 expression and activity in multiple sclerosis. Clin Immunol 2014;150:170–83. 10.1016/j.clim.2013.11.011 24412911

[35] Hashimoto M, Fujii T, Hamaguchi M, Furu M, Ito H, Terao C, et al Increase of hemoglobin levels by anti-il-6 receptor antibody (tocilizumab) in rheumatoid arthritis. PLoS ONE 2014;9: e98202 10.1371/journal.pone.0098202 24878740 PMC4039447

[36] Sallusto F, Geginat J, Lanzavecchia A. Central memory and effector memory T cell subsets: function, generation, and maintenance. Annu Rev Immunol 2004;22:745–63. 15032595 10.1146/annurev.immunol.22.012703.104702

[37] Van Eden W, Van Der Zee R, Van Kooten P, Berlo SE, Cobelens PM, Kavelaars A, et al Balancing the immune system: Th1 and Th2. Ann Rheum Dis 2002;61 Suppl 2:ii25–8. 12379616 10.1136/ard.61.suppl_2.ii25 PMC1766722

[38] Bayes HK, Bicknell S, MacGregor G, Evans TJ. T Helper cell subsets specific for pseudomonas aeruginosa in healthy individuals and patients with cystic fibrosis. PLoS ONE 2014; 9: e90263 10.1371/journal.pone.0090263 24587305 PMC3937364

[39] Uematsu T, Tanaka H, Yamaoka M, Furusawa K. Effects of oral squamous cell carcinoma derived TGF-β1 on CD26/DPPIV expression in T cells. Anticancer Res 2004;24:619–24 15161003

[40] Sánchez-Otero N, Rodríguez-Berrocal FJ, de la Cadena MP, Botana-Rial MI, Cordero OJ. Evaluation of pleural effusion sCD26 and DPP-IV as diagnostic biomarkers in lung disease. Sci Rep 2014;4:3999 10.1038/srep03999 24499783 PMC3915277

[41] Busso N, Wagtmann N, Herling C, Chobaz-Péclat V, Bischof-Delaloye A, So A, et al Circulating CD26 is negatively associated with inflammation in human and experimental arthritis. Am J Pathol 2005;166: 433–42. 15681827 10.1016/S0002-9440(10)62266-3 PMC1602320

