# Correction: CD26 Expression on T Helper Populations and sCD26 Serum Levels in Patients with Rheumatoid Arthritis

**DOI:** 10.1371/journal.pone.0139535

**Published:** 2015-09-28

**Authors:** Oscar J. Cordero, Rubén Varela-Calviño, Tania López-González, Cristina Calviño-Sampedro, Juan E. Viñuela, Coral Mouriño, Íñigo Hernández-Rodríguez, Marina Rodríguez-López, Bruno Aspe de la Iglesia, José María Pego-Reigosa

The last author's name is spelled incorrectly. The correct name is: José María Pego-Reigosa.
